# Association of Common Polymorphisms in *GLUT9* Gene with Gout but Not with Coronary Artery Disease in a Large Case-Control Study

**DOI:** 10.1371/journal.pone.0001948

**Published:** 2008-04-09

**Authors:** Klaus Stark, Wibke Reinhard, Katharina Neureuther, Silke Wiedmann, Kamil Sedlacek, Andrea Baessler, Marcus Fischer, Stefan Weber, Bernhard Kaess, Jeanette Erdmann, Heribert Schunkert, Christian Hengstenberg

**Affiliations:** 1 Klinik und Poliklinik für Innere Medizin II, Klinikum der Universität Regensburg, Regensburg, Germany; 2 Medizinische Klinik II, Universitätsklinikum Schleswig-Holstein, Lübeck, Germany; Leiden University Medical Center, Netherlands

## Abstract

**Background:**

Serum uric acid (UA) levels have recently been shown to be genetically influenced by common polymorphisms in the *GLUT9* gene in two genome-wide association analyses of Italian and British populations. Elevated serum UA levels are often found in conjunction with the metabolic syndrome. Hyperuricemia is the major risk factor for gout and has been associated with increased cardiovascular morbidity and mortality. The aim of the present study was to further elucidate the association of polymorphisms in *GLUT9* with gout and coronary artery disease (CAD) or myocardial infarction (MI). To test our hypotheses, we performed two large case-control association analyses of individuals from the German MI Family Study.

**Methods and Findings:**

First, 665 patients with gout and 665 healthy controls, which were carefully matched for age and gender, were genotyped for four single nucleotide polymorphisms (SNPs) within or near the *GLUT9* gene. All four SNPs demonstrated highly significant association with gout. SNP rs6855911, located within intron 7 of *GLUT9*, showed the strongest signal with a protective effect of the minor allele with an allelic odds ratio of 0.62 (95% confidence interval 0.52–0.75; *p*  =  3.2*10^−7^). Importantly, this finding was not influenced by adjustment for components of the metabolic syndrome or intake of diuretics. Secondly, 1,473 cases with severe CAD or MI and 1,241 healthy controls were tested for the same four *GLUT9* SNPs. The analyses revealed, however, no significant association with CAD or with MI. Additional screening of genome-wide association data sets showed no signal for CAD or MI within the *GLUT9* gene region.

**Conclusion:**

Thus, our results provide compelling evidence that common genetic variations within the *GLUT9* gene strongly influence the risk for gout but are unlikely to have a major effect on CAD or MI in a German population.

## Introduction

Gout is the most frequent inflammatory joint disease in men above 40 years of age [1]. It is characterized by chronic hyperuricemia, usually defined as serum uric acid (UA) levels >7 mg/dL, and recurrent attacks of acute arthritis in one-third of the patients triggered by the release of monosodium urate crystals into the synovial space [2]. In the majority of patients with primary gout, hyperuricemia results from inefficient renal excretion, while in about 10% of cases, hyperuricemia is due to endogenous overproduction of UA [3]. Gout is an increasingly common medical problem with an overall prevalence of at least 1% in Western societies [1]. The reasons for the rise in incidence and prevalence of gout are closely linked to its known association with dietary habits, such as overindulgence in food and alcohol, and with it the intricately connected metabolic syndrome and its components insulin resistance, abdominal obesity, dyslipidemia, and arterial hypertension [4], [5].

The role of hyperuricemia as an independent risk factor for coronary artery disease (CAD) and myocardial infarction (MI) has been discussed controversially for many years since several but not all epidemiologic studies found an independent association of elevated serum UA with cardiovascular disease [6]–[8]. Experimental data to support the hypothesis of a causal relationship between hyperuricemia and CAD are scarce. However, recent genetic data might add further insights into the pathophysiology of hyperuricemia and its possible link to cardiovascular disease. The availability of large-scale genome-wide association (GWA) studies using 500,000 or more single nucleotide polymorphism (SNP) markers equally distributed across the genome, now facilitates identification of susceptibility genes in polygenic and complex phenotypes, e.g. type 2 diabetes or MI [9]–[17]. For hyperuricemia, two very recent GWA studies in an isolated Sardinian population and in a British cohort identified SNPs in the *GLUT9* gene, also known as *SLC2A9*, to significantly influence serum UA levels [18], [19]. GLUT9 is a transmembrane glucose transporter that is highly expressed in liver and kidney [20].

The aims of this study were thus to further assess the association of *GLUT9* polymorphisms in the phenotypic expression of gout and their possible role in susceptibility to CAD, assuming an independent influence of hyperuricemia on the development of cardiovascular disease. For this purpose, we examined two large, separate case-control samples of individuals from the German MI Family Study and included also the results from two CAD/MI GWA studies at the *GLUT9* gene region to analyse potential additive effects.

## Materials and Methods

### Populations

All individuals of this study participated in the German MI Family Study (total *n*  =  7,575). Recruitment process, selection criteria and study details have been reported previously [21], [22]. In brief, we identified families from all parts of Germany with accumulation of premature MI or severe CAD. Control individuals were unaffected spouses of MI family members and had no genetic relationship to cases.

The Ethics committee of the University of Regensburg approved the study protocol and all participants gave their written informed consent at the time of inclusion and again at the time of follow-up investigations. The study was in accordance with the principles of the current version of the Declaration of Helsinki.

### Phenotyping

The baseline home visit was performed by a physician and included a standardized questionnaire, physical examination, and biochemical analyses. A standardized follow-up interview regarding new medical events was carried out by specially trained telephone interviewers after two and five years. Cardiovascular events at study entry and follow-up were validated by reviewing medical records.

The phenotype gout was carefully established using two levels of evidence: 1. medical history readings at time of inclusion, two-year and five-year follow-up interview; and 2. self-reported history of gout. The diagnosis of MI was established according to the MONICA (Monitoring Trends and Determinants in Cardiovascular Disease) diagnostic criteria (http://www.ktl.fi/publications/monica/manual/index.htm). Severe CAD was defined as treatment with percutaneous coronary intervention or coronary artery bypass graft. Resting blood pressure was taken according to MONICA guidelines after participants had been resting in a sitting position [23]. Hypertension was defined as blood pressure ≥140/90 mm Hg or ongoing antihypertensive therapy. Body weight was determined with subjects wearing light clothing. Body mass index (BMI) was calculated as weight in kilograms divided by the square of height in meters. Sampling of serum was carried out from non-fasting individuals. Serum levels of low-density lipoprotein cholesterol (LDL-C) and high-density lipoprotein cholesterol (HDL-C) were measured by standard enzymatic methods. Hypercholesterolemia was defined as LDL-C ≥160 mg/dL or intake of lipid lowering medication. History of diabetes mellitus or intake of antidiabetic medication was used to define type 2 diabetes. Smoking was defined as current or former smoking habit.

### Case-control samples

A total of *n*  =  665 individuals (*n*  =  479 males, *n*  =  186 females) with the diagnosis of gout were selected from the German MI Family Study. All gout cases fulfilled the above mentioned criteria for “gout”. Controls were unrelated individuals from our German MI Family Study who did neither have any indication for gout nor were they medicated with uricostatics or uricosuric agents at any time during follow-up.

We carefully matched the gout-free controls (*n*  =  665) according to age and gender to the gout cases. Further phenotypic details are shown in Table 1.

**Table 1:** Characteristics of gout case and control study sample.

Variable	Gout cases (*n* = 665)	Gout-free controls (*n* = 665)	*p*-Value
Age at inclusion, years (range)^a^	59.9±8.6 (29–84)	59.8±8.4 (31–79)	n. s.
Gender, % male (*n*)^a^	72.0 (479)	72.0 (479)	n. s.
Medication with diuretics, % (*n*)	35.0 (233)	23.3 (155)	<0.0001
MI or severe CAD, % (*n*)	67.5 (449)	65.9 (438)	n. s.
Hypercholesterolemia^b^, % (*n*)	72.6 (483)	69.2 (460)	n. s.
Lipid lowering medication, % (*n*)	48.6 (323)	47.1 (313)	n. s.
LDL-C, mg/dl	150.1±40.2	148.4±39.7	n. s.
HDL-C, mg/dl	49.7±13.8	53.4±15.0	<0.0001
Hypertension^c^, % (*n*)	88.6 (589)	84.5 (562)	n. s.
Antihypertensive therapy, % (*n*)	78.4 (521)	72.8 (484)	n. s.
Systolic blood pressure, mm Hg	140.3±19.0	137.0±18.3	0.0019
Diastolic blood pressure, mm Hg	84.1±10.2	82.0±10.2	0.0003
Type 2 diabetes^d^, % (*n*)	17.7 (118)	10.5 (70)	<0.0001
Smoking^e^, % (*n*)	67.2 (447)	65.1 (433)	n. s.
BMI, kg/m^2^	28.3±3.8	26.9±3.3	<0.0001

Values denote means ± standard deviations unless indicated otherwise. n. s., not significant; CAD, coronary artery disease; MI, myocardial infarction; LDL-C, low-density lipoprotein cholesterol; HDL-C, high-density lipoprotein cholesterol; BMI, body mass index.

a Matching parameter.

b Defined as LDL-C ≥160 mg/dL or intake of lipid lowering medication.

c Defined as blood pressure ≥140/90 mm Hg or ongoing antihypertensive therapy.

d Defined as history of diabetes mellitus or intake of antidiabetic medication.

e Former or current smoking habit.

Futhermore, a large case-control sample was established from the German MI Family Study including 1,473 CAD/MI cases (856 male, 617 female) and 1,241 unrelated CAD/MI-free control individuals (336 male, 905 female). Cardiovascular risk factors and phenotypic details are summarized in Table 2.

**Table 2:** Characteristics of CAD case and control study sample.

Variable	CAD cases (*n* = 1,473)	CAD-free controls (*n* = 1,241)	*p*-Value
Gender, % male (*n*)	58.1 (856)	27.1 (336)	<0.0001
Age at inclusion, years (range)	60.2±8.5 (32–90)	56.4±9.9 (29–84)	<0.0001
Age at first CAD event, years (range)	54.5±9.1 (24–89)	-	-
MI, % (*n*)	75.6 (1,114)	-	-
Gout, % (*n*)	15.5 (228)	8.8 (109)	<0.0001
Hypercholesterolemia^a^, % (*n*)	83.4 (1,228)	29.3 (363)	<0.0001
Lipid lowering medication, % (*n*)	66.7 (982)	38.2 (474)	<0.0001
LDL-C, mg/dl	149.4±42.6	146.1±34.9	0.0313
HDL-C, mg/dl	51.4±13.8	61.6±15.3	<0.0001
Hypertension^b^, % (*n*)	94.4 (1,390)	53.9 (669)	<0.0001
Antihypertensive therapy, % (*n*)	89.3 (1316)	35.0 (434)	<0.0001
Systolic blood pressure, mm Hg	140.0±20.4	132.6±18.2	<0.0001
Diastolic blood pressure, mm Hg	82.6±10.4	81.4±9.8	0.0054
Type 2 diabetes^c^, % (*n*)	11.6 (171)	4.2 (52)	<0.0001
Smoking^d^, % (*n*)	62.7 (924)	48.1 (597)	<0.0001
BMI, kg/m^2^	27.3±3.6	26.5±4.2	<0.0001

Values denote means ± standard deviations unless indicated otherwise. CAD, coronary artery disease; MI, myocardial infarction; LDL-C, low-density lipoprotein cholesterol; HDL-C, high-density lipoprotein cholesterol; BMI, body mass index.

a Defined as LDL-C ≥160 mg/dL or intake of lipid lowering medication.

b Defined as blood pressure ≥140/90 mm Hg or ongoing antihypertensive therapy.

c Defined as history of diabetes mellitus or intake of antidiabetic medication.

d Former or current smoking habit.

### SNP selection and genetic analyses

Four *GLUT9* SNPs were selected from a public reference database (dbSNP, http://www.ncbi.nlm.nih.gov/SNP/) based on the following criteria: (1) evidence for significant association with serum UA levels from two recent publications (rs6855911 and rs7442295) [18], [19], (2) minor allele frequency of >0.10 in a Caucasian population, (3) evidence of validation status, and (4) compatibility with our genotyping platform. To allow determination of the extend of LD beyond the boundaries of the gene, one SNP in the 5′ intergenic region was included.

Genomic DNA was isolated from whole blood samples using the PureGene DNA Blood Kit (Gentra, Minneapolis, MN, USA). DNA samples were genotyped using 5′ exonuclease TaqMan® technology (Applied Biosystems, Foster City, CA, USA). For each genotyping experiment 10 ng DNA was used in a total volume of 5 µl containing 1× TaqMan® Genotyping Master Mix (Applied Biosystems). PCR reaction and post-PCR endpoint plate read was carried out according to the manufacturer's instructions using the Applied Biosystems 7900HT Real-Time PCR System. Sequence Detection System software version 2.3 (Applied Biosystems) was used to assign genotypes applying the allelic discrimination test. Case and control DNA was genotyped together on the same plates with duplicates of samples (15%) to assess intraplate and interplate genotype quality. No genotyping discrepancies were detected. Assignment of genotypes was performed by a person without knowledge of the proband's affection status. Additionally, comparison between genotype calls from TaqMan assays and Affymetrix GeneChip® Human Mapping 500K Array Set were carried out in 288 DNA samples on all four SNPs (rs6855911, rs7442295, rs6449213, and rs12510549). Again, no discrepancies were found. SNPs were mapped to the *GLUT9* gene according to NM_001001290 (position on human genome build 18: chromosome 4; 9,436,948-9,650,970).

**Figure 1:**
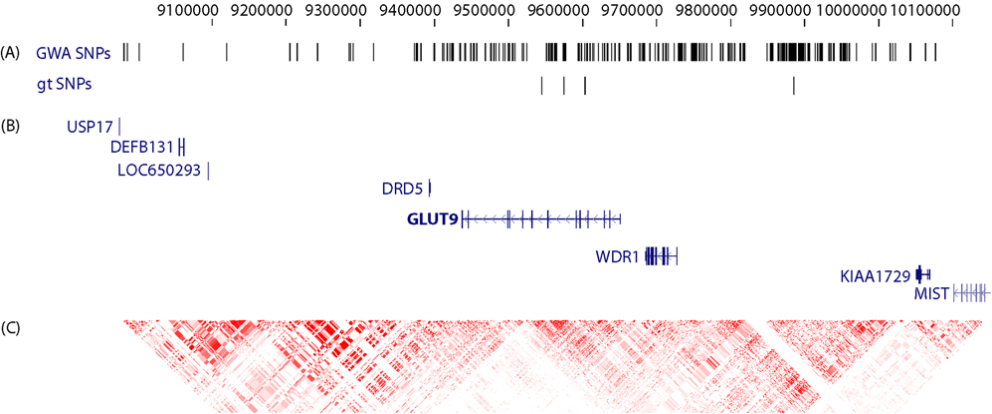
Schematic representation of a ∼1,214 kb region on human chromosome 4p16.1 (position 8,936,948 and 10,150,970; human genome build 18) used for association analyses. Position numbers are given on top. (A) Relative positions of SNPs analyzed in this study. GWA SNPs from WTCCC and Cardiogenics CAD/MI case-control study are shown above the four SNPs genotyped in this work (gt SNPs). From left to right: rs6855911, rs7442295, rs6449213, and rs12510549. (B) Graphic illustration of genes in this genomic region. Relative positions of RefSeq genes with gene names are presented. Exons are depicted as vertical blocks and orientation of genes is indicated by arrows on the horizonal lines representing introns. *GLUT9* gene is highlighted with bold letters. (C) Delineation of LD structure. Pairwise *r* ^2^-values between markers from HapMap phase II release 22 for the CEPH (CEU) sample were calculated and presented. Red denotes perfect LD with *r* ^2^ = 1 and decreasing *r* ^2^-values are shown in brighter color with white representing *r* ^2^ = 0.

Genome-wide data from two recently published screens for polymorphisms associated with CAD or MI were employed to survey the complete *GLUT9* gene region [12]. All successfully genotyped SNPs on the Affymetrix GeneChip® Human Mapping 500K Array Set on chromosome 4 in the *GLUT9* region with 500 kb on either side of the gene (position 8,936,948 and 10,150,970; human genome build 18) were included (Fig. 1).

Data of the HapMap phase II release 22 data were used to assess linkage disequilibrium (LD) patterns of the *GLUT9* gene region [24].

### Statistical analyses

To determine whether the genotypes of cases and controls of all *GLUT9* SNPs deviated from Hardy-Weinberg equilibrium, actual and predicted genotype counts of both groups were compared by χ^2^-test. Differences in allele frequencies between dichotomous traits were calculated employing the same method. Genotypes were coded for both dominant and recessive effects (genotype 22+12 versus 11 and genotype 22 versus 11+12, respectively, with the minor allele coded as 2). The additive genetic model was calculated using Armitage's trend test. Multiple logistic regression analysis was used to examine the association of *GLUT9* SNPs with either phenotype (gout or CAD/MI) allowing adjustment for relevant covariates (Tables 1, 2). Differences in continuous variables between groups were calculated using a two-tailed unpaired *t*-test. Prevalence odds ratios (OR) with their 95% confidence intervals (CI) were reported. A two-sided *p*-value ≤0.05 was considered statistically significant.

Association analyses were performed using JMP IN 7.0.1 (SAS Institute Inc, Cary, NC, USA) and PLINK v1.00 [25]. For haplotype analysis and permutation testing PLINK v1.00 [25] and HaploView v4.0 [26] were employed. Power analysis was carried out using G*Power 3.0.8 [27].

## Results

### Population characteristics

In our first case-control cohort, gout cases (*n*  =  665) were matched for age and gender to control individuals (*n*  =  665). Prevalence of cardiovascular risk factors and cardiovascular disease was high in both, gout cases and gout-free controls. Gout cases were more often treated with diuretics as compared to controls. In addition, in gout cases, the prevalence of type 2 diabetes and increased BMI was higher, whereas in gout-free controls HDL-C levels were elevated. However, we found no significant difference in number of reported MI or CAD events between gout cases (67.5% CAD/MI) and gout-free controls (65.9% CAD/MI). Prevalence of hypercholesterolemia, hypertension and smoking were equally distributed between the two groups (Table 1).

In our second, large case-control sample for CAD/MI the incidence of established cardiovascular risk factors, such as male gender, type 2 diabetes, hypercholesterolemia, hypertension and smoking, as well as increased BMI, was higher in CAD/MI cases (*n*  =  1,473) as compared to controls (*n*  =  1,241) (Table 2). Interestingly, we also found more individuals suffering from gout in our CAD/MI cases compared to CAD/MI-free controls (Table 2).

### Genetic analyses

The cohorts were genotyped for markers rs6855911, rs7442295, and rs6449213 located in *GLUT9* intron 7, 6, and 4, respectively. Additionally, rs12510549, 235 kb upstream of *GLUT9*, was included in the analysis (Table 3). All SNPs fulfilled our criteria of at least 98.5% call rate in all sub-samples. Marker rs6855911 and rs7442295 were reported to be strongly associated with serum UA levels [18]. Pairwise LD analysis showed decreasing *r^2^*-values from rs6855911 to rs12510549 although LD between the four markers remained on an overall high level of *r^2^*≥0.45 (Table 3).

**Table 3:** SNP marker used in analysis.

SNP	Position^b^	Major allele (1)	Minor allele (2)	Function	Pairwise linkage disequilibrium (*r^2^*)^a^		
					rs685591	rs7442295	rs6449213
rs6855911	9,545,008	A	G	Intron 7	-		
rs7442295	9,575,478	A	G	Intron 6	0.815	-	
rs6449213	9,603,313	T	C	Intron 4	0.660	0.809	-
rs12510549	9,885,565	T	C	5′ intergenic	0.450	0.547	0.664

a calculated in gout case-control sample with 1,304 individuals without any missing genotype.

b on chromosome 4 (hg18).

#### Association analysis of *GLUT9* SNPs in the gout case-control sample

Genotype distributions and allele frequencies in gout case-control cohort are shown in Table 4. There was no significant deviation from Hardy-Weinberg equilibrium (HWE) in gout-free controls (*p*  =  0.43, *p*  =  0.45, *p*  =  0.34, and *p*  =  0.21 for rs6855911, rs7442295, rs6449213, and rs12510549, respectively). In contrast, there was a deviation from the HWE in gout cases (*p*  =  0.04, *p*  =  0.01, *p*  =  0.01, and *p*  =  0.02 for rs6855911, rs7442295, rs6449213, and rs12510549, respectively).

**Table 4:** Association analysis results in gout case-control sample.

SNP	Gout case genotypes				Gout-free control genotypes				Genotypic	Allelic	Allelic OR
	11	12	22	MAF	11	12	22	MAF	*p*-value	*p*-value	(95% CI)
rs6855911	423	220	16	0.191	350	255	54	0.275	2.9*10^−7^	3.2*10^−7^	0.62 (0.52–0.75)
rs7442295	449	200	9	0.166	389	230	40	0.235	2.3*10^−6^	8.3*10^−6^	0.65 (0.53–0.78)
rs6449213	469	181	6	0.147	421	207	32	0.205	1.6*10^−5^	8.9*10^−5^	0.67 (0.55–0.82)
rs12510549	459	192	9	0.159	392	238	26	0.221	9.9*10^−5^	5.1*10^−5^	0.67 (0.55–0.81)

MAF: minor allele frequency; numbers of genotypes (11, 12, 22) according to alleles from Table 3.

Strong association with gout was observed for all four markers (Table 4). The highest significance level of *p*  =  2.9*10^−7^ was detected for genotype distribution of marker rs6855911. We found a protective effect of the minor allele with an allelic OR of 0.62 (95% CI 0.52–0.75) (Table 4). Multivariate logistic regression analysis adjusting for BMI, type 2 diabetes, and medication with diuretics even increased the significance of the association (*p*  =  4.6*10^−8^). Hence, effect of marker rs6855911 on susceptibility to gout is independent of common cardiovascular risk factors and several components of the metabolic syndrome.

In addition to allele frequency and genotype distribution, we analyzed additive, dominant and recessive genetic models with no substantial increase in significance (Table 5).

**Table 5:** Results from different genetic models in gout case-control sample.

SNP	Dominant (22+12 vs. 11) *p*-value	Recessive (22 vs. 12+11) *p*-value	Additive *p*-value
rs6855911	4.4*10^−5^	3.1*10^−6^	2.9*10^−7^
rs7442295	5.1*10^−4^	6.6*10^−6^	6.8*10^−6^
rs6449213	2.8*10^−3^	2.0*10^−5^	7.9*10^−5^
rs12510549	2.0*10^−4^	3.4*10^−3^	2.9*10^−5^

According to the high LD between the SNPs, four-marker haplotypes were constructed spanning a 340-kb genomic region. Nine of the 4^2^ possible haplotypes could be observed with only two (AATT and GGCC) having frequencies >0.05 in cases and controls of the gout sample. The most common haplotype AATT (i. e. major allele from each marker) was less frequently present in healthy individuals as compared to patients with gout (frequency in gout-free controls  =  0.691; frequency in gout cases  =  0.780), i. e. representing a risk haplotype, resulting in a *p*-value of 2.2*10^−7^. The second common haplotype (GGCC) codes for a protective haplotype with frequency in controls of 0.183 and frequency in cases of 0.126, respectively (*p*  =  4.5*10^−5^). After adjustment for BMI, type 2 diabetes, and medication with diuretics in a multivariate logistic regression analysis, the association results remained significant (*p*  =  1.3*10^−7^ for AATT and *p*  =  2.9*10^−5^ for GGCC).

Both, single marker and haplotype association analysis remained significant after 100,000 permutations. No permutation χ^2^-value exceeded highest observed χ^2^-value.

Separate analyses in females and males revealed similar odds ratios in allele frequency comparisons but slightly higher significance levels in males (Table 6) than in females (Table 7).

**Table 6:** Association analysis results in male gout case-control sample.

SNP	Gout case genotypes				Gout-free control genotypes				Genotypic	Additive	Allelic	Allelic OR
	11	12	22	MAF	11	12	22	MAF	*p*-value</p>	*p*-value	*p*-value	(95% CI)
rs6855911	297	166	11	0.198	246	192	37	0.280	3.1*10^−5^	2.1*10^−5^	3.0*10^−5^	0.64 (0.51–0.79)
rs7442295	316	151	6	0.172	276	171	28	0.239	1.1*10^−4^	2.5*10^−4^	3.3*10^−4^	0.66 (0.53–0.83)
rs6449213	331	136	5	0.155	298	156	22	0.210	1.0*10^−3^	1.5*10^−3^	1.9*10^−3^	0.69 (0.54–0.87)
rs12510549	324	142	8	0.167	279	176	17	0.223	6.0*10^−3^	1.5*10^−3^	2.2*10^−3^	0.70 (0.56–0.88)

MAF: minor allele frequency; numbers of genotypes (11, 12, 22) according to alleles from Table 3.

**Table 7:** Association analysis results in female gout case-control sample.

SNP	Gout case genotypes				Gout-free control genotypes				Genotypic	Additive	Allelic	Allelic OR
	11	12	22	MAF	11	12	22	MAF	*p*-value	*p*-value	*p*-value	(95% CI)
rs6855911	126	54	5	0.173	104	63	17	0.264	9.4*10^−3^	4.0*10^−3^	2.9*10^−3^	0.58 (0.41–0.83)
rs7442295	133	49	3	0.149	113	59	12	0.223	n. a.	8.5*10^−3^	7.4*10^−3^	0.60 (0.41–0.87)
rs6449213	138	45	1	0.128	123	51	10	0.193	n. a.	0.018	0.016	0.61 (0.41–0.91)
rs12510549	135	50	1	0.140	113	62	9	0.217	n. a.	5.1*10^−3^	5.8*10^−3^	0.59 (0.40–0.86)

MAF: minor allele frequency; numbers of genotypes (11, 12, 22) according to alleles from Table 3.

n. a.: not applicable due to low counts of minor allele for χ^2^.

#### Association analysis of *GLUT9* SNPs in the CAD/MI case-control sample

Our power calculation indicated that at a significance level of 0.05 and with a two-sided alternative hypothesis, our CAD/MI sample with *n*  =  1,473 cases and *n*  =  1,241 CAD-free controls (Table 2) had more than 97% power to detect a significant association with an assumed OR of 1.4 between the tested polymorphisms and the phenotype. HWE criteria for the four genotyped markers were fulfilled in CAD/MI cases and CAD/MI-free controls (*p*>0.4).

No association with CAD was observed for any of the analyzed SNPs (Table 8). To evaluate the impact of a more stringent phenotype definition, we included only MI cases (*n*  =  1,114) as a sub-sample of CAD patients in a separate analysis. Again, no significant difference in allele or genotype frequencies of the four genotyped markers between MI cases and disease-free controls (*n*  =  1,241) was observed (e. g. genotype distribution; rs6855911, *p*  =  0.42; rs7442295, *p*  =  0.27; rs6449213, *p*  =  0.70; rs12510549, *p*  =  0.81). Multivariate logistic regression analysis adjusting for cardiovascular risk factors (age at first manifestation, male gender, type 2 diabetes, hypercholesterolemia, hypertension, smoking, and BMI) both on single SNPs and haplotypes in CAD/MI case-control sample did not reveal positive association results (data not shown). Separate analysis for male and female individuals did not point to gender-specific effects of the four SNPs and CAD/MI in trend test (*p*>0.18).

**Table 8:** Association analysis results in CAD case-control sample.

SNP	CAD case genotypes				CAD-free control genotypes				Genotypic	Allelic	Allelic OR
	11	12	22	MAF	11	12	22	MAF	*p*-value	*p*-value	(95% CI)
rs6855911	808	556	98	0.257	709	455	67	0.239	0.275	0.129	1.10 (0.97–1.25)
rs7442295	884	496	79	0.224	775	415	49	0.207	0.176	0.128	1.11 (0.97–1.26)
rs6449213	939	462	61	0.200	820	374	43	0.186	0.431	0.201	1.09 (0.95–1.25)
rs12510549	901	497	61	0.212	788	397	48	0.200	0.514	0.270	1.08 (0.94–1.23)

MAF: minor allele frequency; numbers of genotypes (11, 12, 22) according to alleles from Table 3.

To gain maximum power for detection of even small effects, we included all appropriate individuals from our German MI Family Study in the CAD/MI case-control sample. This included an overlap of 220 individuals from gout case sample in CAD/MI case sample, 337 individuals from gout control sample in CAD/MI case sample, 108 individuals from gout case sample in CAD/MI control sample, and 217 individuals from gout control sample in CAD/MI control sample, respectively. After an initial round of association testing with CAD/MI, we excluded all overlapping individuals from the analysis. Again, in remaining *n*  =  916 CAD/MI cases compared to *n*  =  916 controls neither SNPs nor haplotypes achieved statistical significance, even after adjustment for age of first manifestation of CAD/MI, male gender, type 2 diabetes, hypercholesterolemia, hypertension, smoking, and BMI (data not shown).

To assess the possibility that other markers located within or close to the *GLUT9* gene could exert influence on susceptibility to CAD or MI, we analyzed the data from two recently conducted GWA studies [12]. Both, from British (WTCCC) and German (Cardiogenics) case-control samples, *p*-values were obtained for association with CAD/MI (WTCCC) and MI (Cardiogenics). A total of 292 SNP markers located in *GLUT9* gene region (214 kb) and a more extended region of 1,000 kb around the *GLUT9* gene were analyzed (Fig. 1). None of the SNPs showed association at a *p*<0.05 with CAD/MI in the WTCCC and Cardiogenics cohorts.

## Discussion

This study investigated the relationship between four common sequence variants and haplotypes within the *GLUT9* gene region with gout and CAD/MI in two German case-control samples. We were led to pursue the *GLUT9* gene because of very recent findings that sequence variants in this gene significantly influence the serum UA levels in Italian and British populations [18], [19].

The prevalence for gout in Germany is 1.4% [28], whereas lifestyle plays an important role on its epidemiology [29]. Hyperuricemia, i. e. high serum UA levels, are associated with the occurrence of gout with some pathophysiologic details still unresolved [30]. The role of gout as a risk factor for CAD and MI was recently demonstrated in epidemiologic data [31]. This prospective study revealed that men with gout had a 59% higher risk of nonfatal MI than men without gout [31].

However, it is still controversial whether hyperuricemia independently predicts cardiovascular events. It is furthermore unclear whether it relates to cardiovascular disease by a complex interaction with several classical risk factors, such as arterial hypertension, obesity, hypercholesterolemia and insuline resistance, all of which are components of the metabolic syndrome [32], [33].

The overall cardiovascular risk is caused by factors like smoking, hypercholesterolemia, hypertension, type 2 diabetes, abdominal obesity, psychosocial factors, physical activity, and nutrition [34]. Additionally, genetic variations modulate not only some of these classical cardiovascular risk factors but act also in a complex manner [35]. Alleles directly influencing susceptibility to CAD or MI in a still unknown fashion were consistently found in three recent GWA studies [10]–[12].

To our knowledge, the present study offers the first case-control association analysis of common *GLUT9* variants with the qualitative phenotypes gout and CAD/MI. We report strong association between all four *GLUT9* SNPs and gout consistently showing a protective effect of the minor alleles with an odds ratio of 0.62. The effect was not influenced by multivariate adjustment for cardiovascular risk factors or metabolic traits and, importantly, intake of diuretic medication pointing to a largely independent genetic effect. Our results are consistent with the findings of Li et al., who demonstrated a significant negative additive effect of about 0.40 mg/dL of *GLUT9* polymorphisms rs6855911 and rs7442295 on serum UA levels resulting from a protective effect of the minor alleles [18]. Therefore, these SNP markers could be helpful for stratification of risk for gout in the general population. Although gout is generally regarded as a disease that is easy to diagnose and has an effective treatment, in less typical cases – particularly in the elderly – the strictly clinical diagnosis becomes less accurate [36]. Misdiagnosing of gout or gouty arthritis as osteoarthritis, rheumatoid arthritis, septic arthritis, or other rheumatic conditions may lead to inappropriate treatment and result in unnecessary morbidity. To support the diagnosis of these atypical presentations genetic testing could be beneficial, help to prevent clinical mismanagement and avoid costs. Maybe, the *GLUT9* gene may even provide a new therapeutic target for hyperuricemia and gout in the future.

For all genotyped markers, we found moderate deviation from HWE in gout cases. Generally, deviations from HWE can point to either a sampling bias or population stratification. Whereas HWE deviations in a control sample suggest the possibility of genotyping errors, HWE deviations in cases can also be the result of true association [37]. We deliberately focused on common sequence variants with MAF between 15 and 20%, as it is more likely that these frequent variants play a role in the general population. Thus, with the MAF being this high, our sample with *n*  =  665 gout cases and *n*  =  665 controls had enough power (>80% with an assumed change of 40% in OR) to detect a true effect.

Additional evidence for a reliable positive association between *GLUT9* SNPs and gout came from analysis of the Framingham SNP Health Association Resource (SHARe) posted on the NCBI dbGaP website at http://www.ncbi.nlm.nih.gov/projects/gap/cgi-bin/study.cgistudy_idphs000007.v2.p1. Here, *n*  =  1,345 adult participants of the largest 310 pedigrees in the Framingham Heart Study were genotyped with the Affymetrix 100K GeneChip [38]. Age, sex and BMI adjusted *p*-values for the phenotype gout (defined as history of gout at any examination cycle) were available (accession: pha001406.1). However, the four SNPs analyzed in our study (rs6855911, rs7442295, rs6449213, and rs12510549) were not included on the Affymetrix 100K GeneChip. For *GLUT9*, marker rs10516195 located in intron 3 near to and in LD with our SNPs (7.6 kb upstream from rs6449213) showed high significance with gout (*p*  =  3.7*10^−8^).

In our study, we found stronger significance between genotyped markers and gout in males than in females. However, odds ratios have the same order of magnitude with a risk reduction of about 40%. It is known that serum UA levels and incidence of gout are higher in males, but tend to equalize with increasing age [39]. In our study, the relatively small female sample size (*n*  =  186 cases and *n*  =  186 controls, respectively) limits the conclusion of gender-specific effects mediated by polymorphisms in *GLUT9* gene on gout.

Also, SNP rs12510549, located 235 kb upstream to *GLUT9* showed significant association with gout in our study. HapMap data describe a large LD block covering two-thirds of *GLUT9* and a large proportion of upstream intergenic sequence, including *WDR1* gene [24] (Fig. 1). It cannot be excluded that the gene encoding WDR1, also known as actin interacting protein 1 (AIP1) contributed at least to a minor portion to the association signals for serum UA levels and gout. Thus far, no functional variants, explaining the effect on serum UA levels in *GLUT9* gene, could be identified by Li et al. [18]. The four SNPs within the *GLUT9* gene region analysed in our study were located in intronic and intergenic regions. A possible effect of a functional variant beyond changes in amino-acid composition of translated protein could lead to altered gene expression, which can be modulated by the concerted action of multiple transcription factors over long distances and, thus, be influenced by multiple SNPs [40]. It could be shown that gene regulatory elements are harboured in non-coding and intergenic regions [41]. The biological function of the *GLUT9* gene is not fully understood yet. It is known, however, that GLUT9 plays an important role as transmembrane sugar transporter and is highly expressed in liver and kidney tubules [20], [42]. Therefore, altered function or expression of GLUT9 in liver may interfere with UA metabolism and increase production of UA, whereas an effect in kidney could lead to reduced renal UA excretion. With elevated serum UA levels, the risk for gout increases considerably, which can be seen in the association analysis of *GLUT9* gene polymorphisms with the phenotype “gout”. However, without the knowledge of serum UA levels in our study, we cannot exclude more direct effects of *GLUT9* gene variants on gout.

In the second case-control analysis, the hypothesis of a causal link of hyperuricemia and elevated risk for CAD or MI was addressed. In a large case-control study, we found no association of the markers in *GLUT9* with CAD/MI. In addition, GWA studies on CAD/MI revealed no significant association signal of SNP markers in the extended *GLUT9* region. Hence, it is unlikely for polymorphisms in *GLUT9* to be major contributors to the risk for CAD or MI. Moreover, a pathophysiologic link between gout and CAD via genetic variations within the *GLUT9* gene appears doubtful. No difference in the prevalence of CAD/MI in the gout case-control sample was observed: 67.5% and 65.9% of the gout cases and gout-free controls, respectively, were affected by CAD/MI (Table 1). However, the reverse was not true for the CAD/MI sample (Table 2). Here, the prevalence of gout and of all components of the metabolic syndrome studied was significantly higher in CAD/MI cases than in CAD/MI-free controls. Due to the study design, we did not dispose of prevalence data for gout until the time point of inclusion and not at the time of the first CAD/MI event. Therefore, prospective analyses on the role of gout on the development of CAD cannot be derived from our data and only descriptive evidence for the relationship between gout and CAD can be given. In addition, it has to be mentioned that our sample provides a special subgroup of the general population and even of CAD patients. This is caused by the inclusion criteria of the German MI Family Study, where particularly high risk individuals from MI families were recruited (index patient suffering from MI before the age of 60 years and further siblings with severe manifestation of CAD/MI agreeing to participate in the study).

Several limitations of our study have to be considered. The gout phenotype was assessed retrospectively from patients records and medical history without knowledge of serum UA levels. However, we combined two levels of evidence, namely medical history readings at time of inclusion and at two-years and five-years follow-up interview together with self-reported history of gout. We only included control individuals without history of gout and without intake of uricostatics or uricosuric agents. The two most confounding effects on association with gout - age and gender - were excluded by carefully matching these parameters between gout cases and gout-free controls. However, in the CAD/MI sample an imbalance on gender existed due to the selection approach of the control individuals in our German MI Family Study (relatives of cases for a disease having a much higher prevalence in males). Therefore, we adjusted for male gender and other cardiovascular risk factors with no increase in significance. Additionally, the two analyzed GWA studies on CAD/MI included a more balanced gender distribution between cases and controls (e. g. in the Cardiogenics MI sample 591 male cases and 284 female cases, respectively, were compared with 813 male controls and 831 female controls, respectively). Here, separate analysis of the four SNPs (rs6855911, rs7442295, rs6449213, and rs12510549) revealed no association with MI, even after adjustment for male gender.

In conclusion, our study demonstrates strong evidence that polymorphisms in *GLUT9* gene are associated with increased susceptibility to gout. Furthermore, these SNPs are unlikely to play a causal role in the pathogenesis of CAD or MI. Additional functional analyses are warranted to further elucidate the role of GLUT9 in the pathophysiology of hyperuricemia and gout.

### Online Resources

Cardiogenics data: http://www.cardiogenics.imbs-luebeck.de/

WTCCC data: http://www.wtccc.org.uk/info/summary_stats.shtml

Framingham SHARe: http://www.ncbi.nlm.nih.gov/projects/gap/cgi-bin/study.cgistudy_id phs000007.v2.p1

PLINK: http://pngu.mgh.harvard.edu/∼purcell/plink/

HaploView: http://www.broad.mit.edu/mpg/haploview/

G*Power: http://www.psycho.uni-duesseldorf.de/abteilungen/aap/gpower3/

HapMap: http://www.hapmap.org/
